# A STAT3-inhibitory hairpin decoy oligodeoxynucleotide discriminates between STAT1 and STAT3 and induces death in a human colon carcinoma cell line

**DOI:** 10.1186/1476-4598-11-12

**Published:** 2012-03-16

**Authors:** Inès Souissi, Patrick Ladam, Jean AH Cognet, Stéphanie Le Coquil, Nadine Varin-Blank, Fanny Baran-Marszak, Valeri Metelev, Remi Fagard

**Affiliations:** 1 INSERM, Unité 978, Bobigny 93017, France; 2 Université Paris 13, UFR SMBH, Bobigny 93017, France; 3 AP-HP, Hôpital Avicenne, Service de biochimie, Bobigny 93009, France; 4 AP-HP, Hôpital Avicenne, Service d'hématologie biologique, Bobigny 93009, France; 5 Moscow State University, Moscow, Russia; 6 CNRS UMR 7244, CSPBAT, Bobigny 93017, France; 7 Université Paris 6, CNRS FRE 3207 ANBioφ, Paris 75005, France

**Keywords:** Hairpin decoy oligodeoxynucleotide (hpdODN), STAT3, STAT1, Colon carcinoma cell line

## Abstract

**Background:**

The Signal Transducer and Activator of Transcription 3 (STAT3) is activated in tumor cells, and STAT3-inhibitors are able to induce the death of those cells. Decoy oligodeoxynucleotides (dODNs), which bind to the DNA Binding Domain (DBD) of STAT3, are efficient inhibitors. However, they also inhibit STAT1, whose activity is essential not only to resistance to pathogens, but also to cell growth inhibition and programmed cell death processes. The aim of this study was to design STAT3-specific dODNs which do not affect STAT1-mediated processes.

**Results:**

New dODNs with a hairpin (hpdODNs) were designed. Modifications were introduced, based on the comparison of STAT3- and STAT1-DBD interactions with DNA using 3D structural analyses. The designed hpdODNs were tested for their ability to inhibit STAT3 but not STAT1 by determining: i) cell death in the active STAT3-dependent SW480 colon carcinoma cell line, ii) absence of inhibition of interferon (IFN) γ-dependent cell death, iii) expression of STAT1 targets, and iv) nuclear location of STAT3 and STAT1. One hpdODN was found to efficiently induce the death of SW480 cells without interfering with IFNγ-activated STAT1. This hpdODN was found in a complex with STAT3 but not with STAT1 using an original in-cell pull-down assay; this hpdODN also did not inhibit IFNγ-induced STAT1 phosphorylation, nor did it inhibit the expression of the STAT1-target IRF1. Furthermore, it prevented the nuclear transfer of STAT3 but not that of IFNγ-activated STAT1.

**Conclusions:**

Comparative analyses at the atomic level revealed slight differences in STAT3 and STAT1 DBDs' interaction with their DNA target. These were sufficient to design a new discriminating hpdODN that inhibits STAT3 and not STAT1, thereby inducing tumor cell death without interfering with STAT1-dependent processes. Preferential interaction with STAT3 depends on oligodeoxynucleotide sequence modifications but might also result from DNA shape changes, known to modulate protein/DNA interactions. The finding of a STAT3-specific hpdODN establishes the first rational basis for designing STAT3 DBD-specific inhibitors.

## Background

STAT3 belongs to the signal transducers and activators of transcription (STATs) family of transcription factors (TFs) [1]. STAT3 is activated in response to several cytokines and growth factors, including IL-6, IL-10, the epidermal growth factor (EGF), and interferon (IFN) α and is also weakly activated in response to other cytokines, including IFNγ in some cellular contexts [2]. Activation of STAT3 involves phosphorylation of tyrosine 705 by cytokine receptor-associated Janus Kinases (JAK); the involvement of the Src and Abl tyrosine kinases as well as EGFR have also been reported [3]. Tyrosine phosphorylation of STAT3 is followed by dimerization through phosphotyrosine-SH2 domain interaction; activated STAT3 enters the nucleus where it stimulates the transcription of its targets, including Cyclin-D1, Survivin, Vegf, C-Myc, Bcl-xL, and Bcl2 [4,5].

STAT3 is a key regulator of cell survival and proliferation [6]. Its constitutive activation has been observed in many human tumors, including colon, breast, lung, pancreas and prostate cancers, melanoma, head and neck squamous carcinoma, multiple myeloma, mantle cell lymphoma, and glioma [7,8]. However, in certain cell types such as PTEN-deficient glioblastoma, STAT3 can become a tumor suppressor [9,10].

STAT1 is another member of the STAT family. It is activated mainly by IFNs α and γ, and plays a major role as a pro-inflammatory, anti-pathogen and anti-proliferative factor [11,12]. Its biological function is thus mostly antagonistic to that of STAT3. Despite their 50% amino acid sequence homology [13], STAT1 and STAT3 are structurally very similar; yet some important differences have been noted in their DBD sequences [14]. Despite its major role as a tumor antagonist, STAT1 can also have functions in cancer cells, as documented in mouse leukemia [15].

Inhibition of STAT3 in tumor cells in which it is constitutively activated leads to cell death [16-18]. This is achieved using either non-specific inhibitors such as curcumin, which also inhibits other transcription factors (NF-κB), or inhibitors specifically designed to inhibit STAT3 through non-covalent binding to the SH2 domain, such as Stattic [19] or STA-21 [20]. Interestingly, these compounds have little effect in cells in which STAT3 is not activated, pointing to STAT3 as a highly valid target to focus on for the design of anti-cancer compounds. However, such compounds are still poorly developed.

TFs activate transcription of their target genes by binding to distinct short DNA consensus motifs. Decoy oligonucleotides (dODNs) containing these consensus motifs can bind the DNA binding domains (DBD) of the TFs and block their activity. dODNs [21] and hairpin dODNs (hpdODNs) [22] have been shown to induce the death of cells in which STAT3 is activated, suggesting that the DBD is another potential target for specific inhibitory compounds. Similarly to double-stranded oligonucleotides that are used to detect active dimers in electrophoretic migration shift assays, STAT3 hpdODNs interact with activated, dimeric STAT3. This interaction impairs the binding of the dimer to importins, resulting in the sequestration of STAT3 in the cytoplasm [23].

Yet, because of the high degree of similarity between STAT3 and STAT1 consensus DNA binding sites, STAT1 competes with activated STAT3 for dODN binding in interferon (IFN) γ-treated cells [22,23], thereby preventing inhibition of active STAT3. Under such conditions the dODN loses its ability to block cell proliferation. In addition, since STAT1 plays a key role in cell death processes [24,25], including caspases expression [26,27] and cooperation with p53 function [28,29], its inhibition by the dODN prevents cell death. Finally, IFNγ being a cell death inducer in several cell types [22,30-32], it is important to design reagents that do not interfere with STAT1, one of its key effectors. Thus, in order to elaborate target-specific anti-cancer compounds, the specificity of hpdODNs to STAT3 needs to be enhanced. It should be noted, however, that in certain cellular contexts STAT1 has been found to be a tumor promoter [15].

The difficulty in designing dODNs recognized by STAT3 but not STAT1 lies in the striking similarity of the consensus DNA sequences of the two TFs [33], in spite of their different cellular functions. Nevertheless, early studies on STAT3/STAT1-discriminating DNA motifs established some sequence preferences that differentiate these TFs [34-39], suggesting possibilities for designing STAT3/STAT1-discriminating dODNs. The notion that discrete nucleotide modifications in target DNA sequences might alter their recognition by closely related TFs is supported by the observation that a single nucleotide change in the κB consensus motif modified NF-κB subunit specificity [40]. Furthermore, DNA recognition by proteins relies in part on DNA shape, known to deviate from the ideal B-conformation. The nature of the nucleotides in the sequence influences conformation and dynamics: for instance, dG:dC stretches confer rigidity [41], pyrimidine/purine steps (particularly T/dA steps) confer flexibility and may also introduce kinks [42,43], and dA:T stretches can have complex configurations [41]. The coordinates from available crystal structures of both STAT1 [44] and STAT3 [45] (in complex with their DNA consensus sequence) were used to analyze their 3D structure using the UCSF Chimera program [46]. Based on the differences found, new hpdODNs were designed and tested for their STAT3/STAT1 discrimination ability by measuring SW480 colon carcinoma cell death and absence of inhibition of STAT1-dependent IFNγ-induced cell death. SW480 cells offer a relevant model since these cells show constitutive activation of STAT3, which is essential for their survival, and they are susceptible to IFNγ-induced cell death, which is a STAT1-dependent process. The newly designed hpdODNs were also compared for their relative binding capacity to STAT1 and STAT3 by performing in-cell pull-downs, and for their ability to prevent nuclear transfer using immunofluorescence.

## Results

### Striking similarities in the interactions of STAT1 and STAT3 with their consensus DNA sequence

**Figure 1:**
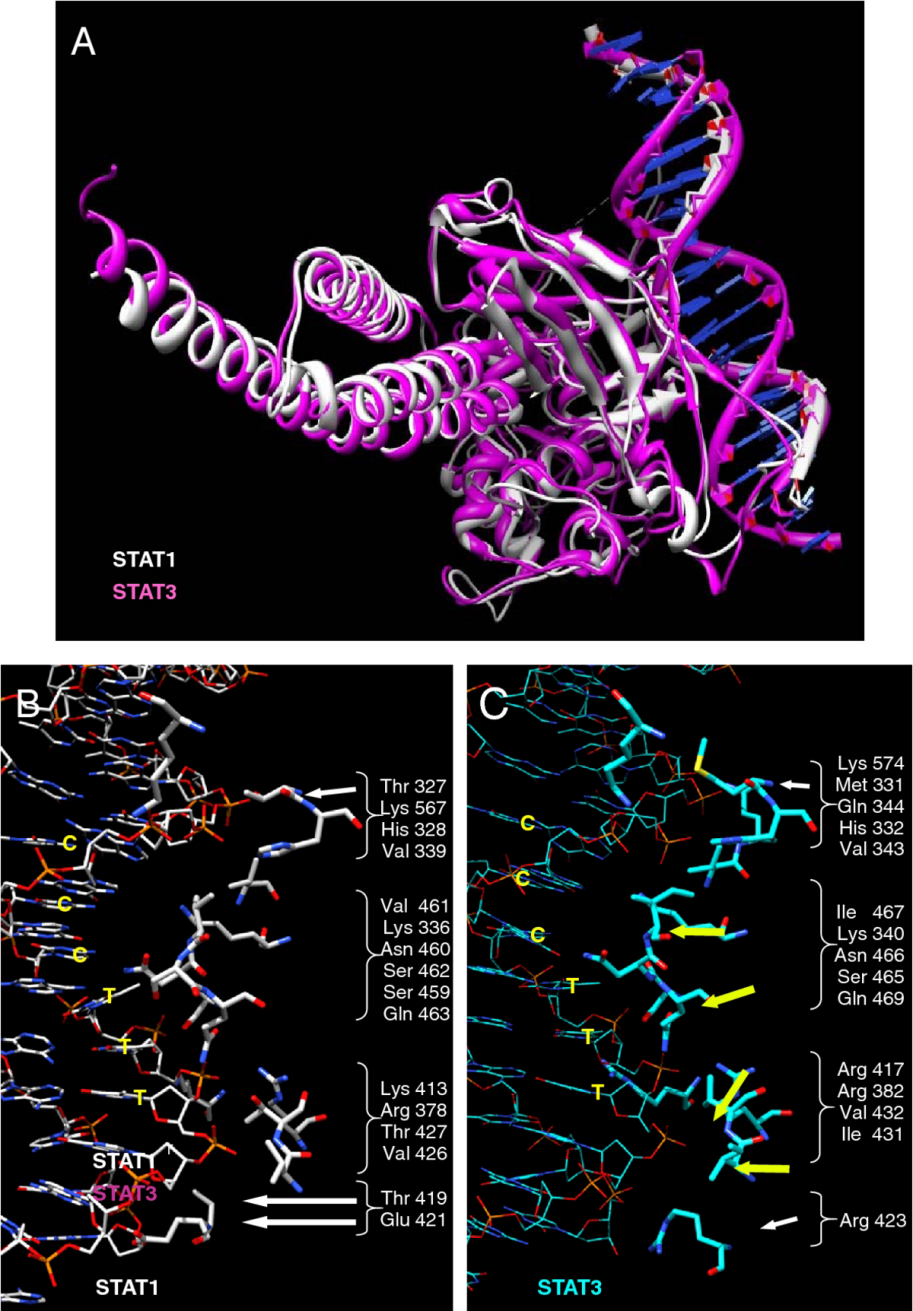
**3D comparison of STAT1 and STAT3 and of their DNA binding region**. **A**: View of the superimposed 3D structure of STAT1 and STAT3 monomers created with the Chimera program, showing the DNA binding region. **B **and **C**: close-up views of the DNA binding regions of STAT1 (B) and STAT3 (C). Amino acids shown are those within a 5 Å distance from DNA; note that Thr 327 did not appear using this selection and was added. Arrows point to amino acids of STAT1 and STAT3 interacting differently with DNA.

**Figure 2:**
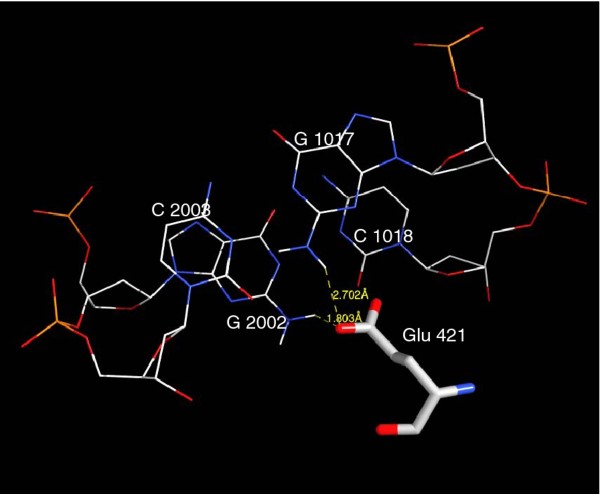
**Location of Glu 421 (STAT1)**. A detailed view of the close interactions of Glu 421 of STAT1 with C 1018, G 1017 and G 2002 in the minor groove of the double helix.

**Figure 3:**
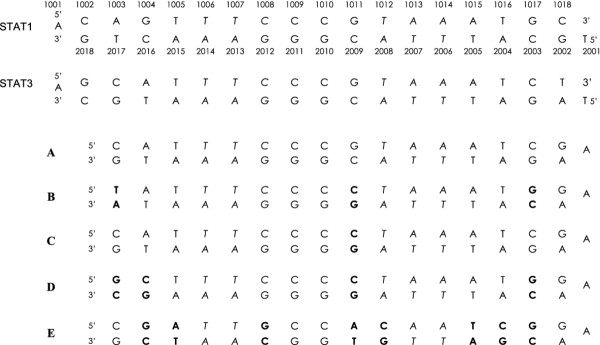
**Sequences used in this study**. The first two sequences depicted are the ones used in the X-ray crystallographic studies of STAT3 [45] and STAT1 [44], based on a STAT3 consensus sequence derived from the c-fos gene promoter [34,35] and a STAT1 consensus sequence derived from the IRF-1 gene promoter [34,35,59]. For clarity, the same numbering (shown here above the STAT1 consensus sequence) was used for all sequences throughout the study. Bases of the consensus sequence are in italic. HpdODN A contains the optimal STAT3 consensus sequence based on the STAT3 binding consensus [34], used in [21,22]. Compared with hpdODN A, hpdODN B is modified at three locations: 1003, 1011 and 1017; hpdODN C is modified at 1011 only; and hpdODN D is modified at 1003, 1004, 1011 and 1017. HpdODN E is a mutated negative control which is modified at 1004, 1005, 1008, 1011, 1012, 1015, 1016 and 1017. Modified bases are in bold.

Comparison of the 3D structures of STAT1 and STAT3 in complex with their oligonucleotide duplexes featuring a consensus DNA sequence using the Chimera program showed that they are highly similar (Figure 1A), with an overall root mean square deviation of 0.63 Å between 317 atom pairs of the backbone. To focus our study on the interaction of the STAT1 and STAT3 DBDs with their consensus DNA sequence, only the amino acids in close contact with the DNA strands were examined. This revealed the striking similarity of STAT1 and STAT3 DNA-interacting amino acids (Figures 1B and 1C). Several differences were noted, however, including: i) Glu 421, unique to STAT1, and located within direct H-bond distance from G 1017 [44], G 2002 and C 1018 (the latter possibly mediated by water) (Figures 1B and 1C; detailed view in Figure 2; see Table 1 for a list of H-bonds and Additional file 1: Table S1 for a list of the interactions of STAT1 and STAT3 with DNA); ii) the peptide backbone of a polar residue of STAT1, Thr 327, and of a hydrophobic residue of STAT3, Met 331, establish H bonds with C 1009 and C 1010 (Figure 1B); iii) a polar amino acid, Thr 419 for STAT1, and a charged amino acid, Arg 423 for STAT3, are identically positioned, facing the backbone of nucleotide 1018 (Figure 1B). To obtain STAT3/STAT1 discriminating sequences, we chose to design hpdODNs, by modifying the original consensus sequences at the specific positions where interactions with STAT1 and STAT3 were found to differ (the consensus sequences and hpdODNs are depicted in Figure 3: for convenience we use the same numbering as in reference [45]).

**Table 1:** Hydrogen bonds involved in the interaction of STAT1 and STAT3 with DNA

STAT1		STAT3	
**Nucleotide involved**	**Residue(s) involved**	**Nucleotide involved**	**Residue(s) involved**

T 1005	Thr 427 (BB/Phos.)	T 1005	Val 432 (BB/Phos.)

T 1006	Lys 413 (BB/Phos.)	T 1006	Ser 465 (SC/Phos.)
	Ser 459 (BB/Phos.)	T 1006	Arg 382 (SC/Phos.)
	Arg 378 (BB/Phos.)	T 1006	Gln 469 (SC/Phos.)
	Ser 459 (BB/Phos.)	T 1006	Ser 465 (SC/Phos.)
	Gln 463 (BB/Phos.)		

T 1007	Asn 460 (BB/Base)	T 1007	Asn 466 ND2(SC/Base)
		T 1007	Arg 417 NH1 (SC/Phos.)

		C 1008	Gln 344 NE2 (SC/Phos.)

		C 1009	Gln 344 N (BB/Phos.)

C 1010	His 328 (SC/Phos.)	C 1010	Lys 340 (SC/Phos.)
		C 1010	His 332 (SC/Phos.)

G 1011	Lys 567(SC/Phos.)		

T 1012	Asn 460 (SC/Base)	T 1012	Asn 466 (SC/Base)

G 1017	Glu 421 (SC/Base)		

C 1018	Thr 419 (BB/Phos.)		
	Thr 419 (BB/Phos.)		

G 2002	Glu 421 (SC/Base)		
	Glu 421 (SC/Base)		

T 2005	Thr 427 (BB/Phos.)	T 2005	Val 432 (BB/Phos.)

T 2006	Lys 413 (SC/Phos.)	T 2006	Ser 465 (SC/Phos.)
	Arg 378 (SC/Phos.)	T 2006	Arg 382 (SC/Phos.)
	Ser 459 (SC/Phos.)	T 2006	Ser 465 (SC/Phos.)
	Arg 378 (SC/Phos.)	T 2006	Gln 469 (SC/Phos.)

T 2007	Asn 460 (SC/Base)	T 2007	Asn 466 (SC/Base)
	Lys 413 (SC/BB)	T 2007	Arg 417 (SC/Phos.)

A 2008	Asn 460 (SC/Base)	A 2008	Gln 344 (SC/Phos.)

		C 2009	Gln 344 (BB/Phos.)

G 2010	His 328 (SC/Phos.)	G 2010	Lys 340 (SC/Base)
		G 2010	His 332 (SC/Phos.)
		G 2010	Lys 340 (SC/Phos.)

G 2011	Lys 567 (BB/Phos.)		

G 2012	Asn 460 (SC/Base)	G 2012	Asn 466 (SC/Base)

A 2013	Asn 460 (SC/Base)		

G 2018	Glu 421 (SC/Phos.)		

The nucleotides that are involved in binding with STAT1 or STAT3 are listed. For clarity, the numbering used is the same as in reference [45] (see Figure **3**). The following abbreviations were used for the atoms involved in hydrogen bonding: phospho-diester (Phos.) or base moiety (Base) for the nucleotides. BB stands for backbone and SC for side-chain for the proteins.

### Nucleotide substitutions provide a hairpin decoy oligonucleotide which can discriminate between STAT1 and STAT3, inhibiting STAT3 in IFNγ-treated cells

**Figure 4:**
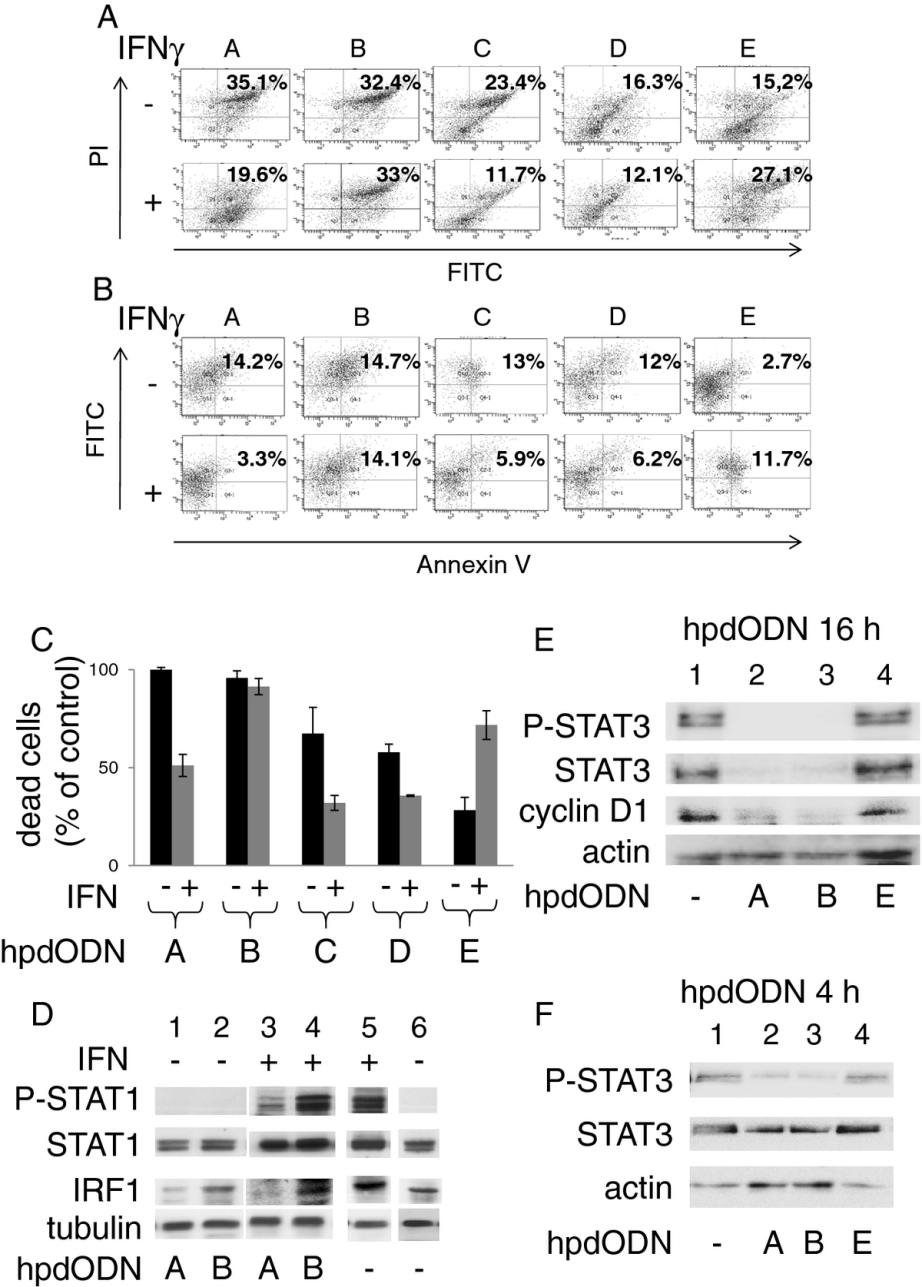
**Comparative cell death induction efficiency of the different hpdODNs in the presence or not of IFNγ in the SW480 cell line**. The efficiencies of hpdODN A, B, C D and E in inducing cell death were compared, in the presence (+) or not (-) of IFNγ. A. Cell death, measured by determining propidium iodide (PI) labeling, induced by hpdODNs A to E (2 μg/ml) alone (-) or in the presence of IFNγ (50 ng/ml 16 h) (+). B. Cell death, measured with flow cytometry for annexin V, induced by hpdODNs A to E (2 μg/ml) alone (-), or in the presence of IFNγ (50 ng/ml 16 h) (+). In A and B, FITC denotes the detection of cells having incorporated the fluorescein-labelled hpdODNs. Note that experimental conditions were such that cell death was not maximal, to allow detection of differences between experiments. Experiments were repeated at least three times, a typical result is shown. C: Histograms from several experiments (at least three per condition) using hpdODNs A to E, obtained by computing PI incorporation and annexin V labelling data (such as those shown in Figures 4A and B). To facilitate comparison, data are presented as% of the control. D: Western blotting showing the phosphorylation of STAT1 on tyrosine ("P-STAT1") and expression level of IRF1 in the absence (lanes 1, 3 and 4) or presence of IFNγ (50 ng/ml, 16 h) (lanes 2, 5 and 6) and in the presence or not of the indicated hpdODNs (2 μg/ml). Anti-phospho-STAT1, anti-STAT1, anti-IRF1 and anti-tubulin antibodies were used. Experiments performed with the mutated control hpdODN E showed no effect (not shown). E: Western blotting showing the inhibition of STAT3 phosphorylation and the reduced expression of cyclin D1 and STAT3 following treatment with hpdODN A (lane 2) or hpdODN B (lane 3), or the mutated hpdODN E (lane 4) (2 μg/ml for 16 h). F: Western blotting showing the inhibition of STAT3 phosphorylation following treatment with hpdODN A (lane 2) or hpdODN B (lane 3), or the mutated hpdODN E (lane 4) (2 μg/ml for 4 h). Experiments were repeated at least three times, one representative experiment is shown.

As previously shown [22,23], the consensus-carrying hpdODN A can efficiently induce the death of cells of the SW480 line (Figure 4A and 4B); but it also inhibits STAT1, thus blocking the STAT1-dependent IFNγ-induced mortality of these cells (Figure 4A, B and 4C) as previously shown [22,23]. hpdODN B was designed by replacing three base pairs in hpdODN A. T replaced dC in position 1003, dC replaced dG in 1011, and dG replaced dC in position 1017 (Figure 3). In the same assay, hpdODN B was found to efficiently induce SW480 cell death but was devoid of any action on IFNγ-induced cell death (Figure 4A and 4B), indicating a preference for STAT3 over STAT1. Features of hpdODN B consist in a stretch of pyrimidines spanning nucleotides 1005 to 1012, a d(TA) step (1003/1004) and a d(TG) step (1016/1017). To analyze the possible effect of only one change in the sequence of hpdODN A, hpdODN C was designed by replacing dG with dC in position 1011 (Figure 3). The killing efficiency of HpdODN C was lower than those of hpdODN A [22,23] and hpdODN B, but in contrast with the latter, it showed a capacity to compete with IFNγ-induced mortality, suggesting that it interacts with STAT1 (Figure 4A and 4B). Next, by placing dG in 1003, dC in 1004, dC in 1011 and dG in 1017 we obtained hpdODN D (Figure 3), which corresponded with a sequence with a marked preference for STAT1 as previously shown by others using a reporter assay [47]. hpdODN D did not induce SW480 cell mortality, but prevented IFNγ-induced killing (Figure 4A and 4B). Finally, hpdODN E, containing a mutated STAT3 binding site (Figure 3) did not induce cell death and did not compete with IFNγ-induced cell death (Figure 4A and 4B). A comparison of the different hpdODNs' IFNγ-independent cell killing efficiency (taking the killing efficiency of hpdODN A as the 100% standard) showed that hpdODN B was twice as efficient as hpdODN A (Figure 4C) and that the control mutated hpdODN E had no effect on cell death (Figure 4C), as previously published [22,23].

### The new STAT3-specific hpdODN B inhibits STAT3 but not STAT1 phosphorylation and inhibits cyclin D1 but not IRF1 expression

To detect the effect of the hpdODNs on STAT3 phosphorylation, IL-6-treated (50 ng/ml) SW480 cells were used. In cells treated with hpdODN B and hpdODN A for 16 h, STAT3 phosphorylation was suppressed (Figure 4E, lanes 2 and 3); the expression of cyclin D1 and of STAT3 itself (STAT3 being its own target) were considerably diminished (Figure 4E, lanes 2 and 3), in agreement with previous observations [22,23]. When cells were treated for 4 h with hpdODNs A and B, phospho-STAT3 was reduced without effect on STAT3 (Figure 4F); the control mutated hpdODN E had no effect (Figures 4E and 4F, lane 4). To confirm that hpdODN B was preferentially inhibiting STAT3 in SW480 cells, the induction of the STAT1-dependent IFNγ target IRF1 was studied. In cells treated with IFNγ (50 ng/ml for 16 h), both phosphorylation of STAT1 and expression of IRF1 increased (Figure 4D, lanes 1 and 2). Treatment with hpdODN A, but not hpdODN B, strongly reduced IRF1 expression (Figure 4D, lanes 3 and 4). In IFNγ-treated cells, the addition of hpdODN A reduced IFNγ-induced IRF1 expression (lane 5) whereas the addition of hpdODN B did not (lane 6). Interestingly, STAT1 phosphorylation on tyrosine was inhibited following treatment with hpdODN A (lane 5) but not with hpdODN B (lane 6). These data indicate that under these experimental conditions hpdODN B does not inhibit STAT1.

### Biotinylated hpdODN B interacts preferentially with STAT3

**Figure 5:**
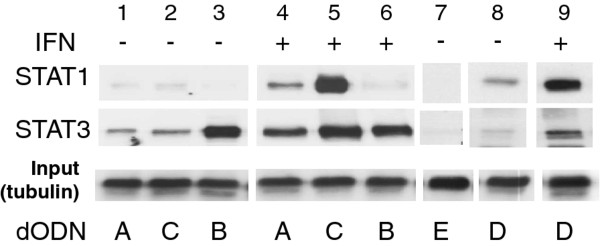
**Specific binding of hpdODN B to STAT3 detected by in-cell pull-down**. Western-blotting of STAT1 and STAT3 bound to biotinylated hpdODNs after transfection and in-cell pull-down with avidine-coated beads. Cells were either not treated (-) (lanes 1, 2, 3 and 7, 8) or treated (+) (lanes 4, 5, 6 and 9) with IFNγ (50 ng/ml, 16 h), and hpdODN A (lanes 1 and 4), hpdODN B (lanes 3 and 6), hpdODN C (lanes 2 and 5) hpdODN D (lanes 8 and 9) and hpdODN E (lane 7) (hpdODNs were at 1 μg/ml for 16 h). Following cell lysis, protein concentration was measured, identical amounts of protein per sample (250 μg) were added; anti-tubulin western blotting from aliquots of the extracts was performed to show identical loading. Binding experiments were repeated at least three times. All lanes are part of the same blot (identical exposure time) and have been separated for clarity.

Binding of STAT3 and STAT1 to hpdODNs has previously been analyzed directly within cells using biotinylated versions of the different hpdODNs [23]. To compare hpdODNs A and B, cells were treated, or not, with IFNγ, transfected with biotinylated hpdODNs, and pull-downs were performed. The pull-down efficiencies of hpdODN A (Figure 5, lanes 1 and 4) and B (Figure 5, lanes 3 and 6) for STAT1 and STAT3 were very different. Indeed, compared with hpdODN A, hpdODN B brought down STAT3 very efficiently, but not STAT1 (Figure 5, lanes 1 and 3), even in IFNγ (50 ng/ml) -treated cells (Figure 5, compare lanes 4 and 6). Furthermore, compared with hpdODN A, hpdODN D, shown to interact preferentially with STAT1, was more efficient in pulling down STAT1 than STAT3 (compare lanes 8 and 9 with lanes 1 and 4). Finally, hpdODN E, a control hpdODN with mutations in the binding consensus, did not bring down either STAT1 or STAT3 (Figure 5, lane 7).

### The new hpdODN B prevents the constitutive nuclear location of STAT3 in SW480 cells, but not that of IFNγ-activated STAT1

**Figure 6:**
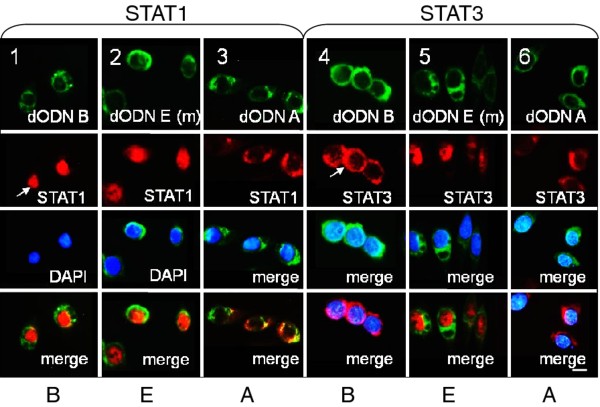
**Differential inhibition of STAT1 and STAT3 nuclear transfer by hpdODN B**. Immunofluorescence analysis of STAT1 and STAT3 nuclear transfer in SW480 cells incubated with fluorescein-labelled hpdODNs A, B and E (green). STAT1 nuclear transfer was induced by treating cells with IFNγ (50 ng/ml, 16 h) (columns 1, 2 and 3). Columns 3 and 6: hpdODN A; columns 1 and 4: hpdODN B; columns 2 and 5: hpdODN E (mutated control). All hpdODNs were at 1 μg/ml. STAT1 and STAT3 were detected using specific antibody and a second rhodamine-coupled antibody (red), nuclei (blue) were stained using DAPI and detected under UV light; the different images were merged to examine the subcellular location of the fluorescein-tagged hpdODNs (green) and STAT1 or STAT3 (red). Arrows point to nuclear STAT1 and cytoplasmic STAT3 in IFNγ-treated and hpdODN B-treated cells. Size bar: 10 μm. One representative experiment out of at least three is shown.

HpdODNs A and B were further compared for their ability to prevent the nuclear translocation of STAT3 and STAT1 in SW480 cells (treated or not with IFNγ) using immunofluorescence. Treatment of the cells with hpdODN A prevented the nuclear translocation of both STAT3 and STAT1 (for STAT1 nuclear translocation, IFNγ-treated cells were used) (Figure 6), as previously shown [22,23]. Treatment with hpdODN B prevented the nuclear translocation of STAT3 only, and not that of IFNγ-activated STAT1 (Figure 6, columns 1 and 4, see arrows), confirming its discriminative capacity. Notably, the control mutated hpdODN E had no effect on the subcellular location of either STAT3 or STAT1, which both remained nuclear (Figure 6, columns 2 and 5).

## Discussion

A new hairpin decoy oligonucleotide (hpdODN) carrying STAT3's DNA binding consensus sequence was designed following 3D analysis of protein/DNA interaction and shown to induce the death of STAT3-dependent tumor cells without interfering with STAT1, a key effector of cell death. In this paper, 3D structural analyses of the protein/DNA interaction of STAT1 and STAT3 demonstrated their high similarity, confirming previous reports [13]. These 3D analyses served as a basis for the design of new sequences with base substitutions. The new sequences were tested for their ability to induce cell death in an IFNγ-sensitive [22,23,48-51], active-STAT3-dependent colon carcinoma cell line [22]. This enabled the design of the STAT3-specific hpdODN labeled here as hpdODN B. The ability of hpdODN B to discriminate between STAT1 and STAT3 was assessed by: i) its ability to kill cells without interfering with IFNγ-induced cell death; ii) its ability to inhibit STAT3 targets, including cyclin D1, iii) the absence of inhibition of IFNγ-induced STAT1 phosphorylation and IRF1 expression, iv) its lack of interaction with STAT1 in pull-down assays and iv) its inability to inhibit IFNγ-induced STAT1 nuclear location. Indeed, hpdODN A treatment, but not hpdODN B treatment, reduced STAT1 phosphorylation, probably by impairing nucleocytoplasmic shuttling as previously suggested [22,23]. Nevertheless, despite its ability to discriminate between STAT1 and STAT3, hpdODN B probably has a residual affinity for STAT1, as shown by low detection of STAT1 in pull-down assays and the fact that cell death induction by hpdODN B and IFNγ are not additive.

The STAT3/STAT1-discriminating hpdODN was obtained by replacing key nucleotides that 3D analyses had shown to be in the vicinity of amino acids of the DBD that distinguish the two STATs; the similarity of their DNA consensus sequences, despite their different functions, has been recognized for some time [33]. Examination of the nucleotide modifications that led to STAT1/STAT3-discriminating hpdODN B showed that they are compatible with previous in vitro DNA-binding studies, such as the preference for T at 1003 and 1005, dC at 1010 and dA at 1015 of STAT3 [36-38]. The fact that T at 1003 does not favor STAT1 binding is also in agreement with the earlier suggestion that "selection for a dG:dC base pair at position 7 *(here: 1017) *is likely to involve Glu 421 (*of STAT1*) which can accept hydrogen bonds from guanine in the minor groove" [44]. This has also been noted by others [39]. Finally, altered recognition by a TF following single nucleotide changes has been previously shown, for instance with NF-κB subunit recognition of κB [40]. One notable property of the hpdODN B is its dissymmetry. A symmetric version (with dA replacing dG in 1017) was tested and is apparently not different from hpdODN B (not shown). Intriguingly, although the preference of hpdODN D for STAT1 was expected from previous data showing its STAT1-specific binding [47], its basis is not clear and may rest upon properties beyond nucleotide sequence such as DNA shape. The shape and flexibility of DNA strands are known to be influenced by their nucleotide content; here the 8-pyrimidine stretch in hpdODN B may confer a higher flexibility than hpdODN A and may account for a differential interaction with STAT3 Arg 423 and STAT1 Glu 421. In fact, the molecular dynamics studies which describe a scissor-like molecular movement upon DNA binding for STAT3, but not for STAT1 [13] suggest that the flexibility of the DNA target may play a role in binding and therefore underly the preference of hpdODN B for STAT3. It may also account for the greater sensitivity of STAT3 to an intact palindromic structure compared to STAT1, as previously stated [38]. Protein binding itself can affect DNA bending, as shown with the high-affinity target of the papillomavirus E2 [52]. Nevertheless, despite its efficiency, the precise mechanism whereby the hpdODN B discriminates between STAT1 and STAT3 in cells is not understood. Changes in DNA shape may play a role in the preferential recognition of hpdODN B by STAT3; co-factors may also be involved in DNA recognition by STAT3, and might associate more efficiently when hpdODN B is used. The process might also be more complex than mere differential DNA binding: STAT1 and STAT3 are reciprocally regulated [2,53-55] and the relative abundance of their active forms may itself play a key role in biological responses, as previously discussed [56]. Another level of complexity arises from the fact that in cells in which STAT3 has been suppressed, IFNγ-activated STAT1 induces the expression of mitogenic STAT3-targets [57,58]. Furthermore, STAT1 and STAT3 form heterodimers, whose function has not been elucidated to date. In this respect, quantification of the relative amounts of STAT1 and STAT3 bound to the hpdODNs A and B may help understand the complex interaction of these TFs. Preliminary experiments that are underway suggest a difference in heterodimer content. Therefore, it is possible that hpdODN B functions in cells by tilting the active STAT1/active STAT3 balance toward STAT1, thereby inducing cell death.

## Conclusions

By combining 3D molecular interaction analysis and direct screening in cells, this work allowed the design of an hpdODN that can selectively inhibit STAT3 but not STAT1. The efficacy and potential of this approach resides in the direct testing of modified hpdODNs in cells, analyzing processes that depend on STAT3 (cell survival) or STAT1 (IFNγ-mediated death). These hpdODNs represent a basis for elaborating STAT3 DBD-specific low molecular weight compounds with anti-cancer properties.

## Material and methods

### Computer analysis of STAT3 and STAT1

The PDB (Protein Data Base) files for STAT1 (1BF5) [44] and STAT3 (1BG1) [45] were downloaded and analyzed using Chimera [46]. The STAT1 and STAT3 crystals used in the X-ray diffraction studies were proteins complexed with oligonucleotide duplexes featuring a consensus DNA sequence [34,35,59] (see Figure 3). To compare the STAT1 and STAT3 DBDs in a complex with their DNA consensus sequences, the missing complementary strand of the STAT3-bound oligonucleotide [45] was reconstructed through crystal symmetry operations.

### Decoy oligonucleotides

#### The STAT3 decoy ODNs used were

RHN-(CH_2_)_6_-CATTTCCCGTAAATCGAAGATTTACGGGAAATG-(CH_2_)_6_-NHR (STAT3 decoy hpdODN), derived from the serum-inducible element of the human c-fos promoter [34] (hpdODN A) and previously used in the lab [22,23].

RHN-(CH_2_)_6_-TATTTCCCCTAAATGGAACATTTAGGGGAAATA-(CH_2_)_6_-NHR (hpdODN B); RHN-(CH_2_)_6_-CATTTCCCCTAAATCGAAGATTTAGGGGAAATG-(CH_2_)_6_-NHR (hpdODN C); RHN-(CH_2_)_6_-GCTTTCCCCTAAATGGAACATTTAGGGGAAAGC-(CH_2_)_6_-NHR (hpdODN D); and the following mutated hpdODN as a negative control:

RHN-(CH_2_)_6_-CGATTGCCACAATCGGAACGATTGTGGCAATCG-(CH_2_)_6_-NHR (hpdODN E) (where R was either: hydrogen, fluorescein or biotin). The addition of fluorescein or biotin, followed by high-performance liquid chromatography, were carried out by the manufacturer (Eurogentec, Seraing, Belgium) using in-house protocols. The hairpin sequence GAA, previously shown to confer stability and nuclease resistance [60,61], was included in the dODNs. In the hpdODNs, the hairpin motif was built and incorporated in the X-ray structure using the BCE (Biomolecular Chain Elasticity) approach [62]; this showed that the hairpin did not interfere with the DBD-DNA interaction (not shown).

### Cell culture and reagents

SW480 (colon adenocarcinoma) cells were grown in DMEM (Invitrogen, Lifetechnologies, Cergy-Pontoise, France), supplemented with 10% FCS (Lonza, Levallois-Perret, France), 100 U/ml penicillin, 10 μg/ml streptomycin (GibcoBRL), 1 mM sodium pyruvate (GibcoBRL), MEM vitamins (100x, Invitrogen) and 5 μg/ml plasmocin (Cayla InvivoGen, Toulouse, France). Sodium orthovanadate (100 μM) was from Fischer (Illkirch, France). Interferon γ (50 ng/ml) was from Promocell (GmbH, Heidelberg, Germany) or Sigma-Aldrich (Montigny-le-Bretonneux, France).

### Transfections

Cells were grown in 4-well plates to a density of 0.25 × 10^6 ^cells/ml. When the cells reached 50-60% confluence, they were transfected with the different STAT3 hpdODNs or the control hpdODN (2 μg/ml, corresponding to 400 nM) into 150 μL of DMEM medium (without SVF) combined with polyethyleneimine (PEI, reference 408727, average MW: 25000, Sigma-Aldrich), with an hpdODN/PEI ratio of 1:1. For immunocytochemistry, liposomes prepared as previously described [23] were used (at 2 μg of cationic lipid). After 6 h at 37°C in a humidified 5% CO_2 _incubator, the cells were placed in fresh serum-containing medium. Cells were examined after 48 h in the humidified incubator.

### Flow cytometry and cell viability

To measure cell death, cells were resuspended in annexin V-binding buffer, incubated with 5 μL of propidium iodide (PI, BD Pharmingen, Morangis, France) and subjected to flow cytometry analysis, using a FACS Canto II Flow Cytometer (BD). To enable selective analysis of the cells that had incorporated the various hpdODNs, fluorescein-labelled hpdODNs were used. Fluorescein-labelled cells were analyzed for PI incorporation (red channel) or annexin V labelling (APC, red channel). A cell death index was established through computation of averages.

### Gel electrophoresis, western blotting

Cells were washed in Phosphate-Buffered Saline (PBS), lysed in sodium dodecyl sulfate (SDS) sample buffer (50 mM Tris-HCl pH 6.8 (Bio-Rad, Marnes-la-Coquette, France), 2% SDS (Sigma-Aldrich), 20% glycerol (Prolabo, Fontenay-sous-Bois, France), 1 mM sodium vanadate (Na_3_VO_4_, Labosi, Elancourt, France), 1 mM dithiothreitritol (DTT) (Merck, Fontenay-sous-Bois, France) and 0.01% bromophenol blue (Sigma-Aldrich)), sonicated and stored at -70°C. Proteins (50 μg) were separated on SDS polyacrylamide gels (PAGE) (10%) and transferred onto nitrocellulose membranes; membranes blocked with dry skimmed milk (5%) in Tris Buffered Saline (TBS) were incubated with antibody overnight at 4°C. Anti-phospho-STAT1, anti-STAT1 and anti-STAT3 (1:1000) (Cell Signaling, Ozyme, Montigny-le-Bretonneux, France), anti-cyclin D1 and anti-IRF1 (1:1000) (Santa Cruz, Tebu-bio, Le Perray en Yvelines, France) were used. Blots were washed in TBS with Tween (0.05%) (TBS-T), incubated with peroxidase-coupled goat anti-mouse (Santa Cruz) or goat anti-rabbit (Upstate, Ozyme) secondary antibody (1:20,000), washed in TBS-T and revealed by chemiluminescence (LumiGLO reagent and peroxide; Cell Signaling) and autoradiography (X-Omat R film; Kodak). When necessary, membranes were stripped with Blot Restore Kit (Chemicon International, Millipore, Saint-Quentin-en-Yvelines, France) and reprobed with anti-tubulin (Cell Signaling) or anti-actin (Santa Cruz) antibody to ensure equal loading of the gels. Prestained molecular weight standards (Fermentas, Saint-Rémy-lès-Chevreuse, France) were used.

### Oligodeoxynucleotide pull-down

For in-cell hpdODN pull-down assays, cells were transfected with the biotinylated hpdODNs (1 μg/ml, 6 h), as described under oligonucleotide transfection, and then lysed in cell lysis buffer (1% NP40, 50 mM Hepes, pH 7.6, 140 mM NaCl) containing salmon sperm DNA (1 μg/assay). Protein concentration was measured in the samples. Extracts (250 μg of protein per tube) were recovered on avidin-sepharose beads (50 μL) (NeutrAvidin, Pierce); beads were incubated for 30 min at 4°C in binding buffer (1% NP40, 50 mM Hepes, pH 7.6, 140 mM NaCl). After washing with binding buffer, complexes were eluted in SDS sample buffer, separated on SDS-PAGE (8%), and subjected to immunoblotting using anti-STAT1 or anti-STAT3 antibodies (Cell Signaling) and processed as above.

### Immunocytochemistry

Cells were grown at 50-60% confluence in 8-well plates (Lab-Tek, Nunc, Rochester, USA) to a density of 10^5 ^cells/ml. Cells were transfected with fluorescein-labelled hpdODNs, incubated (6 to 16 h), washed in PBS, fixed with 3.7% formaldehyde for 15 min, permeabilized in 0.1% Triton X-100 for 15 min and incubated in 5% FCS-0.1% Tween- PBS for 1 h. Cells were stained with anti-STAT3 or anti-STAT1 antibody (Cell Signaling) (1:100) for 2 h, then stained with an Alexa fluor 546-labeled secondary antibody (Invitrogen) (1:200) for 90 min. Cells, counterstained with 4', 6'-diamidino-2-phenylindole (DAPI), were mounted onto glass slides with Vectashield (Vectorlabs, Clinsciences, Montrouge, France). Fluorescence images were acquired using a Zeiss Axioplan 2 Deconvolution microscope (Carl Zeiss, Le Pecq, France) and analyzed with Metafer4 (Metasystems, Altlussheim, Germany).

## Competing interests

The authors declare that they have no competing interests.

## Authors' contributions

IS, PL, VM, SLC, JAHC and RF made substantial contributions to conception, design, and data acquisition, analysis and interpretation; NVB and FBM were involved in revising the manuscript for important intellectual content; and IS, PL, VM, JAHC, SLC, NVB, FBM and RF gave final approval of the version to be published. All authors read and approved the final manuscript.

## Supplementary Material

Additional file 1 **Table S1 List of the contacts between STAT1, STAT3 and DNA**. Contacts were evaluated using the "Find clashes/contacts" routine in Chimera with the default parameters (cut off = -0.4 Å and allowance for potentially hydrogen-bonded pairs = 0.0 Å). These values allow the van der Waals radii in atom pairs to be taken into account rather than interatomic distances alone. Click here for file
