# Supplementary material for: A STAT3-inhibitory hairpin decoy oligodeoxynucleotide discriminates between STAT1 and STAT3 and induces death in a human colon carcinoma cell line
Source: Mol Cancer. 2012 Mar 16;11:12. doi: 10.1186/1476-4598-11-12 (PMC3325846; doi:10.1186/1476-4598-11-12)
Supplement: Additional file 1 — Table S1 List of the contacts between STAT1, STAT3 and DNA. Contacts were evaluated using the "Find clashes/contacts" routine in Chimera with the default parameters (cut off = -0.4 Å and allowance for potentially hydrogen-bonded pairs = 0.0 Å). These values allow the van der Waals radii in atom pairs to be taken into account rather than interatomic distances alone. [file 1476-4598-11-12-S1.DOC]

Additional file S1

Table

List of STAT1- and STAT3-DNA contacts.

| STAT1 | | | STAT3 | | |
| --- | --- | --- | --- | --- | --- |
| Nucleotide involved | Number  of  contacts | Residues involved | Nucleotide involved | Number of contacts | Residues involved |
| T 1005 | 4, 10, 2 | Val 426, Thr 427, Gln 463 | T 1005 | 3, 6, 1 | Ile 431, Val 432, Gln 469 |
| T1006 | 2, 4, 9, 1, 5 | Arg 378, Lys 413, Ser 459, Asn 460, Gln 463 | T 1006 | 3, 10, 1, 1 | Arg 382, Ser 465, Asn 466, Gln 469 |
| T 1007 | 3, 1, 1 | Lys 413, Ser 459, Asn 460 | T 1007 | 1, 3, 2 | Arg 417, Ser 465, Asn 466 |
|  |  |  | C 1008 | 2 | Gln 344 |
| C 1009 | 1, 5 | Thr 327, Val 339 | C 1009 | 2, 5, 3 | Met 331, Val 343, Gln 344 |
| C 1010 | 5, 3, 1, 3 | Thr 327, His 328, Lys 336, Val 461 | C 1010 | 4, 3, 2 | His 332, Lys 340, Ile 467 |
| G 1011 | 1, 1 | Asn 460, Lys 567 | G 1011 | 2, 2 | Ile 467, Lys 574 |
| T 1012 | 5, 1 | Asn 460, Ser 462 | T 1012 | 4 | Asn 466 |
| C 1018 | 10, 8 | Thr 419, Glu 421 | T 1018 | 5 | Arg 423 |
| G 2002 | 3 | Glu 421 |  |  |  |
| T 2005 | 6, 8, 2 | Val 426, Thr 427, Gln 463 | T 2005 | 3, 6, 1 | Ile 431, Val 432, Gln 469 |
| T 2006 | 3, 4, 1, 4 | Arg 378, Lys 413, Thr 427, Ser 459 | T 2006 | 3, 1, 9, 1, 1 | Arg 382, Val 432, Ser 465, Asn 466, Gln 469 |
| T 2007 | 4, 1, 3 | Lys 413, Ser 459, Asn 460 | T 2007 | 1, 3, 2 | Arg 417, Ser 465, Asn 466 |
| A 2008 | 1 | Asn 460 | A 2008 | 2 | Gln 344 |
| C 2009 | 3 | Val 339 | C 2009 | 2, 5, 3 | Met 331, Val 343, Gln 344 |
| G 2010 | 6, 1 | Thr 327, His 328 | G 2010 | 4, 4, 2 | His 332, Lys 340, Ile 467 |
| G 2011 | 2 | Lys 567 | G 2011 | 2, 2 | Ile 467, Lys 574 |
|  |  |  | G 2012 | 5 | Asn 466 |
| A 2013 | 1 | Asn 460 |  |  |  |
|  |  |  | C 2018 | 5 | Arg 423 |

Contacts have been evaluated using the “contacts” routine in chimera with the default parameters (cutoff = –0.4 Å and allowance for potentially hydrogen-bonded pairs = 0.01 Å).

These values allow to take the Van der Waals radii in atom pairs into account instead of the simple interatomic distances.
